# Influence of Broiler Age on the Apparent Metabolizable Energy of Cereal Grains Determined Using the Substitution Method

**DOI:** 10.3390/ani12020183

**Published:** 2022-01-13

**Authors:** Mahmoud M. Khalil, Mohammad Reza Abdollahi, Faegheh Zaefarian, Peter V. Chrystal, Velmurugu Ravindran

**Affiliations:** 1 Monogastric Research Center, School of Agriculture and Environment, Massey University, Palmerston North 4442, New Zealand; M.Abdollahi@massey.ac.nz (M.R.A.); F.Zaefarian@massey.ac.nz (F.Z.); V.Ravindran@massey.ac.nz (V.R.); 2 Complete Feed Solutions, Howick, Auckland 2145, New Zealand; Peter@completefeeds.co.nz

**Keywords:** age, apparent metabolizable energy, grains, substitution, broilers

## Abstract

**Simple Summary:**

Knowledge of the metabolizable energy content of cereal grains is critical for their economical and sustainable use and precise poultry feed formulation. The current practice in the feed industry is to use the apparent metabolizable energy (AME) or nitrogen-corrected AME (AMEn) values of ingredients from prediction equations or reference tables, which have been estimated using (5-week-old birds). Several factors, including age, ingredient type, and methodology, can affect the AMEn value of ingredients in poultry. Currently, there are no data available on the age effect, from hatch to 6 weeks of age, on the AMEn of grains in broilers. The aim of the present study was to investigate the influence of age on the AMEn of wheat, sorghum, barley, and corn from hatching to day 42 using the substitution method. The results showed that the age influence on the AMEn of cereal grains was grain dependent. In wheat and sorghum, AMEn was influenced by age, while the AMEn of barley and corn were unaffected. Poultry nutritionists might need to consider age-dependent AME or AMEn values for some grains in feed formulations.

**Abstract:**

The present study investigated the influence of broiler age on the AMEn of wheat, sorghum, barley, and corn using the substitution method at six different ages (days 7, 14, 21, 28, 35, and 42). A corn-soybean meal basal diet was formulated and, the test diets were developed by replacing (*w*/*w*) 300 g/kg of the basal diet with wheat, sorghum, barley, or corn. Bird age influenced (*p* < 0.001) the AMEn of wheat and sorghum but had no effect (*p* > 0.05) on those of barley and corn. The AMEn of wheat increased with age (*p* < 0.001) from 12.53 MJ/kg DM in week 1 to 14.55 MJ/kg DM in week 2, then declined subsequently, but no linear or quadratic responses were observed. The AMEn of sorghum demonstrated a quadratic response (*p* < 0.05), increasing from 12.84 MJ/kg DM in week 1 to 13.95 MJ/kg DM in week 2, and then plateauing to week 6. Overall, the present results suggest that the effect of broiler age on the AMEn varies depending on the grain type. The current data suggest that the application of age-dependent AME or AMEn of wheat and sorghum will lead to more precise feed formulations.

## 1. Introduction

Available energy in feed or feed ingredients for poultry can be measured by different systems, with the apparent metabolizable energy (AME) [1], despite its limitations [2,3], being the commonly accepted and extensively used system.

Three methods, namely, direct, substitution (or difference), and regression, have been used to determine the AME of ingredients for poultry. In each method, the excreta can be collected by total collection, which is the preferred method, or partial collection (marker method) using the ratio of an indigestible marker present in diet and excreta. Each method has its own merits and drawbacks, and the main difference between these methods is how the test ingredients are included in the assay diets [3].

The direct method, which is used mainly to estimate the AME of cereal grains [4], is based on feeding the test ingredient as the sole source of energy in the assay diet. A limitation of this method is that it cannot be used for longer feeding periods due to the nutritionally unbalanced assay diet. Moreover, it cannot be used for ingredients with poor palatability [5,6].

The substitution method is used to determine the AME of poorly palatable ingredients or of those containing high protein content or anti-nutrients. This method requires formulating two sets of diets, a basal (or reference diet) and a test diet, which is developed by replacing a portion of the basal diet with the test ingredient [7,8]. Because the reference diet is a nutritionally balanced diet, this method overcomes the main limitation of the direct method. However, the substitution method suffers from some disadvantages in that the AME of the test ingredient can be influenced by the composition of the basal diet, the assumption of additivity, and the inclusion level of the test ingredient [3,9].

Another alternative for estimating the AME of ingredients is the regression method. In this method, a basal diet and several test diets in which the basal diet is replaced by at least two levels of the test ingredient are fed. The energy value of individual diets is compared to the corresponding inclusion level of the test ingredient, and extrapolation of energy to the equivalency of 100% inclusion of test ingredient predicts the AME [10,11]. However, this method is seldom used due to the errors associated with the calculations and the high cost involved in running the in vivo trials.

Estimation of AME of a feed ingredient, therefore, can vary depending on the methodology [9,12,13]. In our previous study [4], the direct method was employed to determine the AME of wheat, sorghum, barley, and corn at different ages (1–6 weeks) of broiler chickens. It was found that broiler age has a substantial influence on the AME and nitrogen-corrected AME (AMEn) of all grains, but the effect varied depending on the grain type. The aim of the present study was to determine the impact of age on AMEn of the same batch of cereal grains (wheat, sorghum, barley, and corn) as those used in a previous study [4] for broiler chickens using the substitution method.

## 2. Materials and Methods

The experiment was conducted according to the New Zealand Revised Code of Ethical Conduct for the use of live animals for research, testing, and teaching and approved by the Massey University Animal Ethics Committee.

### 2.1. Ingredients

Four cereal grains (wheat, sorghum, barley, and corn) were obtained from a local commercial supplier. The proximate and nutrient composition of the cereal grains is presented in Table 1. The wheat and sorghum samples were of Australian origin, and corn and barley were sourced from New Zealand. All grains were ground in a hammer mill to pass through a screen size of 3.0 mm.

### 2.2. Diets, Birds, and Housing

A total number of 1260 day-old male broilers (Ross 308) were obtained from a local hatchery and raised on floor pens in an environmentally controlled room until assigned weekly to the experimental treatments. Except for the 1–7 d age group, birds were fed broiler starter mini pellets (230 g/kg crude protein and 12.56 MJ/kg AMEn) until d 21 and finisher pellets (207 g/kg crude protein and 13.0 MJ/kg AMEn) from d 21 to 35 (Table 2). At the beginning of each week (d 1, 8, 15, 22, 29, and 36), a new batch of birds was selected randomly from the floor pens, weighed individually, and allocated to cages so that the average bird weight per cage was similar. For each cereal grain, the assay diet was fed to six replicate cages of broilers during the six periods, namely week 1 (d 1–7), week 2 (d 8–14), week 3 (d 15–21), week 4 (d 22–28), week 5 (d 29–35) or week 6 (d 36–42). Each replicate cage housed 10 birds during week 1, 8 birds during week 2, and 6 birds during weeks 3 to 6 post hatch.

The AME was determined using the substitution method. In this method, a corn-soybean meal basal diet was formulated (Table 2), and then the four test diets were developed by replacing (*w*/*w*) 300 g/kg of the basal diet with one of the cereal grains [3]. Diets were mixed in a single-screw paddle mixer (Bonser Engineering Co. Pty. Ltd., Merrylands, Australia), then pelleted using a pellet mill (Model Orbit 15; Richard Sizer, Kingston-upon-Hull, UK) capable of manufacturing 180 kg of feed/h and equipped with a die ring with 3 mm holes and 35 mm thickness.

### 2.3. Determination of Metabolizable Energy

The AME was determined using the total excreta collection procedure [1]. During each week, diets were fed for 7 d, with the first 3 d serving as an adaptation period. The feed intake (FI) and total excreta output for each replicate cage were recorded over the last 4 consecutive d of the assay. Daily excreta collections were pooled within a replicate cage, mixed in a blender, and sub-sampled. Sub-samples were lyophilized (Model 0610, Cudon Engineering, Blenheim, New Zealand), and dried excreta samples were ground to pass through a 0.5 mm sieve and stored in airtight plastic containers at 4 °C pending analysis. The diet and excreta samples were analyzed for dry matter (DM), gross energy (GE), and nitrogen (N).

### 2.4. Chemical Analysis

Dry matter was determined using standard procedures (Method 930.15) [14]. Ash was determined by a standard procedure (Method 942.05) [14] using a muffle furnace at 550 °C for 16 h. Nitrogen was determined by combustion (Method 968.06) [14] using a carbon nanosphere-200 carbon, N, and sulfur auto analyzer (rapid MAX N exceed, Elementar, Donaustraze, Hanau, Germany). The crude protein content was calculated as N × 6.25. The crude fat was determined by the Soxtec extraction procedure (Method 2003.06) [14] using (Soxtec System HT 1043 Extraction Unit, Höganäs, Sweden). Starch was measured using a Megazyme kit (Method 996.11) [14] based on thermostable α-amylase and amyloglucosidase [15]. The neutral detergent fiber (Method 2002.04) [14] was determined using Tecator Fibertec™ (FOSS Analytical AB, Höganäs, Sweden). For minerals, samples were ashed, and then calcium and phosphorus were determined calorimetrically following digestion with HCl (Method 968.08D) [16]. Gross energy was determined by an adiabatic bomb calorimeter (Gallenkamp Autobomb, Weiss Gallenkamp Ltd., Loughborough, UK) standardized with benzoic acid.

### 2.5. Calculations

All data were expressed on a DM basis, and the AME was determined using the following formula:

AME_Diet_ (MJ/kg) = [(FI × GE_Diet_) − (Excreta output × GE_Excreta_)]/FI

The AME of the cereal grains was then calculated using the following formula:

AME_Grain_ (MJ/kg) = [AME of test grain diet − (AME of basal diet × 0.70)]/0.30

Nitrogen retention, as a percentage of intake, was determined as follows:

N retention (%) = 100 × [((FI × N_Diet_) − (Excreta output × N_Excreta_))/(FI × N_Diet_)]

The AMEn was then calculated by correction for zero N retention by assuming 36.54 KJ per g N retained in the body as described by Titus et al. [17].

### 2.6. Statistical Analysis

The data for each grain were analyzed separately by one-way ANOVA using the general linear models procedure of the SAS (version 9.4; SAS Institute Inc., Cary, NC, USA). Cages served as the experimental unit. Significant differences between means were separated by the least significant difference test. The data were subjected to orthogonal polynomial contrasts using the general linear models procedure of SAS [18] to examine whether the responses to increasing bird age were of linear or quadratic nature. The significance of the effects was declared at *p* ≤ 0.05.

## 3. Results

The influence of broiler age on the retention of DM and N, AME and AMEn of wheat is summarized in Table 3.

**Figure 1:**
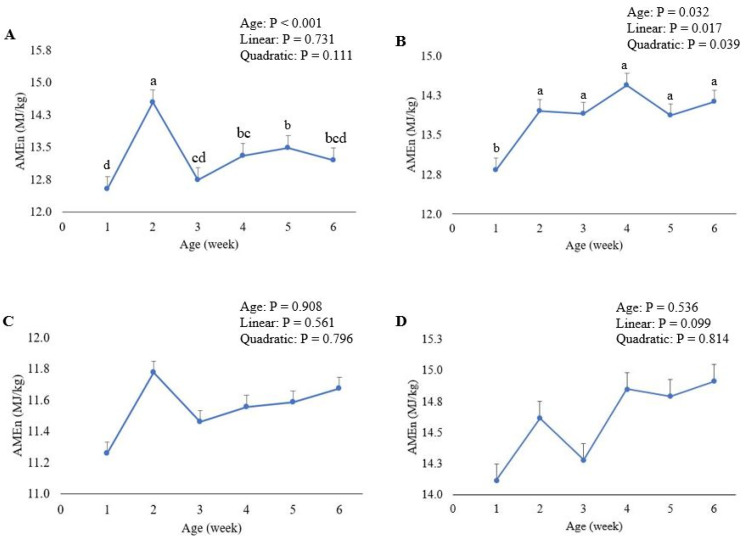
Effect of broiler age on the nitrogen-corrected apparent metabolizable energy (AMEn) for wheat (**A**), sorghum (**B**), barley (**C**), and corn (**D**); mean ± standard error. ^a–d^ Values with different superscripts differ significantly (*p* < 0.05).

The retention of both DM and N showed a linear response (*p* < 0.001) with advancing age, with the retentions decreasing as the birds grew older. The highest DM and N retentions were recorded in weeks 1 and 2. Although the birds’ age did not exhibit any linear or quadratic response (*p* > 0.05; Figure 1A), the AMEn of wheat was observed to increase (*p* < 0.001) from 12.53 MJ/kg DM in week 1 to 14.55 MJ/kg DM in week 2, then declined in following weeks compared to week 2.

The influence of broiler age on the retention of DM and N, AME and AMEn of sorghum is presented in Table 4. Dry matter and N retentions decreased (*p* < 0.001) linearly with the advancing age of birds. The DM retention declined from 77.8% in week 1 to 74.7% in week 6, and the highest N retention of 70.9% was recorded in week 1, declining to 58.1% in week 6. The AMEn of sorghum increased quadratically (*p* < 0.05) with advancing age, from 12.84 MJ/kg DM in week 1 to 13.95 MJ/kg DM in week 2, then plateaued up to week 6 (Figure 1B).

The influence of broiler age on the retention of DM and N, AME and AMEn of barley is presented in Table 5. The retention of DM and N showed linear decreases (*p* < 0.001) with advancing age. The birds retained the highest DM and N in week 1 and the lowest in week 6. Broiler age had no influence (*p* > 0.05) on the AME or AMEn of barley (Figure 1C).

The retention of DM and N, AME and AMEn of corn measured in broilers at different ages are presented in Table 6. The DM retention declined linearly (*p* < 0.001) from 80.3% in week 1 to 76.9% in week 6. A similar trend was observed for N retention, wherein N retention decreased linearly (*p* < 0.001) as the birds grew older from 76.8% in week 1 to 63.1% in week 6. The AME and AMEn of corn were unaffected (*p* > 0.05) by the age of broilers (Figure 1D).

## 4. Discussion

The objectives of the present study were to investigate whether (i) the AMEn of commonly used cereal grains measured by the substitution method is influenced by the age of broilers and (ii) the AMEn estimates of cereals are comparable to those determined using the direct method in our previous study [4].

When the direct method was employed in the AME assay [4], the highest DM and N retention were observed in week 1 and then declined with age for all cereal grains. Somewhat similar trends were observed for retention values in the current AME assay using the substitution method. The highest retention of DM and N were recorded in weeks 1 and 2 and declined thereafter as the birds grew older. These findings are similar to those of Lopez and Leeson [19], who showed that the retention of N in a corn-soybean meal diet declined as broilers grew older, especially after 28 d of age. Aderibigbe et al. [20] similarly reported significant reductions in the retention of DM and N in a corn-soybean meal diet from 1 to 42 d of age of broiler chickens. Yang et al. [21] reported that the advancing age of broilers significantly decreased the N retention of cereal-based diets from 68.8% at 7 d of age to 60.9% at 35 d of age. The observed age-related reductions in the N retention in the current study, and the previous ones, are to be expected, reflecting surplus N from increasing feed consumption and decreasing needs of N for growth [22].

In our previous study [4], following the direct method, the highest AMEn values were observed in week 1 for all four cereal grains and then declined with age. In the present study, with the substitution method, the trends were exactly the opposite. In general, the lowest AMEn values were recorded in week 1 for all cereal grains (statistically significant for wheat and sorghum and numerically for barley and corn) and increased thereafter. Published data on the influence of broiler age on the AME of cereals are limited, and all available data relate to complete diets. Current findings agree with previous studies in broilers fed complete practical diets, where the use of energy-yielding nutrients improved with age [23,24]. Batal and Parsons [24] showed that the AMEn of a corn-soybean meal diet increased with age (from 13.33 MJ/kg at 7 d to 14.35 MJ/kg at 14 d) and then plateaued after 14 d of age. However, in a subsequent study by the same authors [25], no differences were observed in the AMEn of a corn-soybean meal diet between 7 and 14 d of age. Thomas et al. [26] showed that the AMEn of wheat- and corn-based diets increased between d 7 (11.06 and 12.28 MJ/kg, respectively) and d 14 (13.24 and 13.01 MJ/kg, respectively), with no further change between 14 and 21 d of age. Aderibigbe et al. [20] observed that the AMEn of a corn-soybean meal diet increased from 13.6 to 13.8 MJ/kg between 11 and 21 d of age, then plateaued until 42 d of age.

In diets containing adequate levels of protein, AME is a function of the use of lipids and starch. Available data on fat and starch digestion patterns lend support to the increase in AME with age. Tancharoenrat et al. [27], investigating several fat sources, found that the total tract fat digestibility was low in week 1 and increased with advancing age. A similar observation was reported by Lessire et al. [28], who examined the influence of age on the fat digestibility and AME of beef tallow. It was found that the apparent fat digestibility and AME of beef tallow increased by 8.5% and 4.3%, respectively, between weeks 2 and 6. Scheele et al. [29] also revealed that the apparent digestibility of animal fat increased after the second week post hatch, and the AME increased by 1.0 MJ/kg between weeks 2 and 4. Batal and Parsons [24] showed that the apparent digestibility of fat in a corn-soybean meal diet increased with advancing age from 59% at week 1 to 74% at week 2 post hatch. These researchers attributed the increase in AMEn to the increase in fat digestibility with the advancing age of broilers. Svihus [30] indicated that there is a strong correlation (*r* = 0.984) between the AME and digestibility of starch, the main source of energy in cereal-based diets. Hatchlings can digest starch rapidly due to high activity levels and accumulation of starch-degrading endo-enzymes such as α-amylase and disaccharidase in the pancreas during the latter stages of embryonic development [31,32]. Akiba and Murakami [33] stated that the activity of amylase increased by 10% between 1 and 21 d post hatch. Noy and Sklan [34] also reported that the secretion of amylase was low at 4 d post hatch and increased by 100 folds at 21 d post hatch; however, there was no difference in starch digestibility between 4 and 21 d of age. Uni et al. [35] found that starch digestibility of a corn-soybean meal diet increased from 90% at d 4 to 95% at d 14 of age. Similar increases in starch digestibility with advancing broiler age have been reported by Batal and Parsons [24] and Zelenka and Ceresnakova [36].

It is acknowledged that the data from the current study (substitution method) cannot be statistically compared with those from our previous study determined using the direct method [4]. However, since the same samples of the four cereals were evaluated in both studies, a general inference of the effect of methodology could be made. Consistent with previously published research, the AME estimates were influenced by the methodology [12,13,37]. There were two key differences between the findings of the two studies. First, the AMEn of cereal grains determined by the substitution method was lower, with average differences ranging from 2.0 MJ/kg at week 1 to 0.45 MJ/kg at later ages. The average difference between the AMEn determined by direct and substitution methods was highest for barley (1.42 MJ/kg) followed by sorghum (1.21 MJ/kg), and the lowest difference was recorded for wheat (0.29 MJ/kg). The greatest difference in AMEn estimates between the methods was recorded for the first week post hatch, which could be related to the differences in feed intake between the two methods. Higher feed intake is recognized to have a negative influence on dietary AMEn [38,39]. Second, trends of AMEn with advancing broiler age differed between the two methods. In the direct method, the AMEn was greater in week 1 and then declined, whereas the AMEn was lower in week 1 and then increased in the substitution method. Such a divergence was unexpected and has not been reported previously. Overall, these findings add further complications to the existing inconsistencies in AMEn determination assays [3].

Possible explanations for the variation in AME estimates among methodologies lie primarily in the differences in assay diet composition and calculation methods. Only one previous study has compared the substitution and direct methods. Lockhart et al. [12] reported that the AME of wheat was lower when measured by the direct method (12.91 vs. 13.09 MJ/kg). Veluri and Olukosi [13], comparing the substitution and regression methods, found that the assay method can influence the AME and that methodology differences should be considered in comparisons across studies. Lee and Kong [37] found that the AME of barley measured by the direct method was lower than that measured by the regression method (11.42 vs. 12.43 MJ/kg). The observed difference was attributed to the high inclusion level of barley in the direct method, leading to greater β-glucan content and digesta viscosity, hence decreasing the digestion and absorption of nutrients [40,41]. Olukosi [9] reported that AMEn of barley measured by the regression method was 2.96 MJ/kg greater than that of the substitution method (10.97 vs. 8.01 MJ/kg), but the AMEn of corn was not influenced by the methodology, suggesting that the influence of the methodology is ingredient dependent. However, Lee and Kong [37] observed no significant differences for the AME of wheat when measured by the direct vs. regression method.

## 5. Conclusions

The current findings, along with those from previous studies, demonstrate that the effects of age and methodology are relevant in the determination of AMEn of cereal grains. The influence of the age of birds on the AMEn of cereal grains was grain dependent. While AMEn of wheat and sorghum were influenced by age, the AMEn of barley and corn were unaffected. The direct method yielded higher AMEn estimates than the substitution method, but this does not necessarily make the method that provided greater values more robust. Importantly, current findings question the validity of using single AME or AMEn values for feed ingredients in broiler diet formulations across different ages.

## Figures and Tables

**Table 1:** Proximate, carbohydrate, and mineral composition of the tested cereal grains (g/kg; as received basis).

Parameter	Wheat	Sorghum	Barley	Corn
DM ^a^	899	909	925	909
Ash	18.4	15.5	16.1	20.5
Nitrogen	19.7	17.0	20.0	12.9
Protein	123.1	106.3	125.0	80.6
Fat	18.5	32.6	22.0	32.4
Starch	532	606	499	590
NDF ^a^	103	62.2	90.1	83.1
Ca ^a^	0.21	0.10	0.19	0.17
P ^a^	3.51	2.89	2.65	2.47
GE ^a^ (MJ/kg)	16.18	16.68	16.69	16.25

^a^ Ca, calcium; DM, dry matter; GE, gross energy; NDF, neutral detergent fiber; P, phosphorus.

**Table 2:** Composition (g/kg, as fed basis) of the basal diet used in the apparent metabolizable energy assay and of pre-assay diets fed to broiler starters (d 1 to 21) and finishers (d 22 to 35).

Ingredient	Basal Diet	Starter Diet	Finisher Diet
Corn	604.4	574.2	660.0
Soybean meal, 460 g/kg	338.1	381.4	295.7
Soybean oil	14.2	8.8	13.6
Dicalcium phosphate	15.8	10.7	8.2
Limestone	10.4	11.3	9.9
L lysine HCl	3.7	2.0	1.9
DL methionine	3.1	3.3	3.0
L threonine	2.0	1.0	0.7
L valine	0.7	-	-
Sodium chloride	1.0	2.5	2.5
Sodium bicarbonate	3.9	2.7	2.5
Trace mineral premix	1.0	1.0	1.0
Vitamin premix ^1^	1.0	1.0	1.0
Choline chloride 60%	0.7	-	-
Ronozyme HiPhos (Phytase)	-	0.1	0.1

^1^ Vitamin and trace mineral premix supplied the following per kilogram of diet: antioxidant, 100 mg; biotin, 0.2 mg; calcium pantothenate, 12.8 mg; vitamin D3 (cholecalciferol), 2400 IU; cyanocobalamin, 0.017 mg; folic acid, 5.2 mg; menadione, 4 mg; niacin, 35 mg; pyridoxine, 10 mg; vitamin A (trans-retinol), 11100 IU; riboflavin, 12 mg; thiamine, 3.0 mg; vitamin E (dl-α-tocopheryl acetate), 60 IU; choline chloride, 638 mg; Co, 0.3 mg; Cu, 3.0 mg; Fe, 25 mg; I, 1 mg; Mn, 125 mg; Mo, 0.5 mg; Se, 200 µg; Zn, 60 mg.

**Table 3:** Influence of broiler age on the retention (% of intake) of dry matter (DM) and nitrogen (N), apparent metabolizable energy (AME; MJ/kg DM), and nitrogen-corrected AME (AMEn; MJ/kg DM) of wheat ^1^.

Age (Week)	DM Retention	N Retention	AME	AMEn
1	77.6	76.2	13.30	12.53
2	81.6	76.4	15.28	14.55
3	73.6	64.5	13.35	12.75
4	75.3	65.7	13.84	13.31
5	75.4	65.7	14.03	13.48
6	74.3	59.6	13.76	13.20
SEM ^2^	0.66	1.23	0.273	0.240
Orthogonal polynomial contrast, P≤				
Linear	0.001	0.001	0.667	0.731
Quadratic	0.271	0.157	0.274	0.111

^1^ Each value represents the mean of six replicates. The number of birds per replicate cage was 10 (week 1), 8 (week 2), and 6 (weeks 3–6). ^2^ Pooled standard error of the mean.

**Table 4:** Influence of broiler age on the retention (% of intake) of dry matter (DM) and nitrogen (N), apparent metabolizable energy (AME; MJ/kg DM), and nitrogen-corrected AME (AMEn; MJ/kg DM) of sorghum ^1^.

Age (Week)	DM Retention	N Retention	AME	AMEn
1	77.8	70.9	13.32	12.84
2	78.0	68.2	14.38	13.95
3	75.9	65.7	14.39	13.90
4	77.2	65.2	14.86	14.45
5	75.6	63.6	14.29	13.87
6	74.7	58.1	14.48	14.13
SEM ^2^	0.61	1.25	0.344	0.321
Orthogonal polynomial contrast, P≤				
Linear	0.001	0.001	0.047	0.017
Quadratic	0.468	0.376	0.043	0.039

^1^ Each value represents the mean of six replicates. The number of birds per replicate cage was 10 (week 1), 8 (week 2), and 6 (weeks 3–6). ^2^ Pooled standard error of the mean.

**Table 5:** Influence of broiler age on the retention (% of intake) of dry matter (DM) and nitrogen (N), apparent metabolizable energy (AME; MJ/kg DM), and nitrogen-corrected AME (AMEn; MJ/kg DM) of barley ^1^.

Age (Week)	DM Retention	N Retention	AME	AMEn
1	75.8	74.2	11.98	11.26
2	75.6	72.2	12.46	11.78
3	72.8	65.7	12.09	11.46
4	72.9	64.3	12.06	11.56
5	72.5	65.3	12.15	11.59
6	71.3	59.3	12.24	11.67
SEM ^2^	0.63	1.03	0.360	0.325
Orthogonal polynomial contrast, P≤				
Linear	0.001	0.001	0.906	0.561
Quadratic	0.429	0.278	0.972	0.796

^1^ Each value represents the mean of six replicates. The number of birds per replicate cage was 10 (week 1), 8 (week 2), and 6 (weeks 3–6). ^2^ Pooled standard error of the mean.

**Table 6:** Influence of broiler age on the retention (% of intake) of dry matter (DM) and nitrogen (N), apparent metabolizable energy (AME; MJ/kg DM), and nitrogen-corrected AME (AMEn; MJ/kg DM) of corn ^1^.

Age (Week)	DM Retention	N Retention	AME	AMEn
1	80.3	76.8	14.68	14.12
2	79.8	73.1	15.09	14.62
3	77.2	69.6	14.79	14.28
4	78.4	69.8	15.33	14.85
5	77.8	68.3	15.24	14.80
6	76.9	63.1	15.38	14.92
SEM ^2^	0.53	1.12	0.366	0.359
Orthogonal polynomial contrast, P≤				
Linear	0.001	0.001	0.152	0.099
Quadratic	0.233	0.930	0.879	0.814

^1^ Each value represents the mean of six replicates. The number of birds per replicate cage was 10 (week 1), 8 (week 2), and 6 (weeks 3–6). ^2^ Pooled standard error of the mean.

## Data Availability

All available data are incorporated in the manuscript.
